# ITI-Signals and Prelimbic Cortex Facilitate Avoidance Acquisition and Reduce Avoidance Latencies, Respectively, in Male WKY Rats

**DOI:** 10.3389/fnbeh.2014.00403

**Published:** 2014-11-21

**Authors:** Kevin D. Beck, Xilu Jiao, Ian M. Smith, Catherine E. Myers, Kevin C. H. Pang, Richard J. Servatius

**Affiliations:** ^1^Neurobehavioral Research Laboratory, VA New Jersey Health Care System, East Orange, NJ, USA; ^2^Stress and Motivated Behavior Institute, Rutgers – New Jersey Medical School, Rutgers Biomedical and Health Sciences, Rutgers – The State University of New Jersey, East Orange, NJ, USA; ^3^Department of Neurology and Neurosciences, Rutgers – New Jersey Medical School, Rutgers Biomedical and Health Sciences, Rutgers – The State University of New Jersey, Newark, NJ, USA; ^4^Veterans Biomedical Research Institute, East Orange, NJ, USA

**Keywords:** prelimbic cortex, infralimbic cortex, lever-press avoidance, safety signals, conditioned inhibitor, anxiety vulnerability

## Abstract

As a model of anxiety disorder vulnerability, male Wistar-Kyoto (WKY) rats acquire lever-press avoidance behavior more readily than outbred Sprague-Dawley rats, and their acquisition is enhanced by the presence of a discrete signal presented during the inter-trial intervals (ITIs), suggesting that it is perceived as a safety signal. A series of experiments were conducted to determine if this is the case. Additional experiments investigated if the avoidance facilitation relies upon processing through medial prefrontal cortex (mPFC). The results suggest that the ITI-signal facilitates acquisition during the early stages of the avoidance acquisition process, when the rats are initially acquiring escape behavior and then transitioning to avoidance behavior. Post-avoidance introduction of the visual ITI-signal into other associative learning tasks failed to confirm that the visual stimulus had acquired the properties of a conditioned inhibitor. Shortening the signal from the entirety of the 3 min ITI to only the first 5 s of the 3 min ITI slowed acquisition during the first four sessions, suggesting the flashing light (FL) is not functioning as a feedback signal. The prelimbic (PL) cortex showed greater activation during the period of training when the transition from escape responding to avoidance responding occurs. Only combined PL + infralimbic cortex lesions modestly slowed avoidance acquisition, but PL-cortex lesions slowed avoidance response latencies. Thus, the FL ITI-signal is not likely perceived as a safety signal nor is it serving as a feedback signal. The functional role of the PL-cortex appears to be to increase the drive toward responding to the threat of the warning signal. Hence, avoidance susceptibility displayed by male WKY rats may be driven, in part, both by external stimuli (ITI signal) as well as by enhanced threat recognition to the warning signal via the PL cortex.

## Introduction

Anxiety disorders are a product of experience and underlying vulnerabilities (Merikangas et al., 1999). Since avoidance is a prime symptom of all anxiety disorders, avoidance susceptibility can be considered a vulnerability factor for maladaptive coping and anxiety disorder development (Kashdan et al., 2006). The underlying source of avoidance susceptibility is unknown, but it may involve inherent differences in the perception of threat versus safety. Some avoidance models have utilized discrete stimulus cues to represent oncoming noxious stimuli (threat) and/or periods when aversive stimuli are never present (i.e., safety). Individuals with anxiety disorders commonly do not react to signals associated with safety in the same manner as controls (Rachman, 1984; Grillon, 2002; Schmidt et al., 2006; Lohr et al., 2007; Jovanovic et al., 2010), and regions of prefrontal cortex that have been implicated in the perception of threat versus safety in animals may also be involved in the expression of anxiety disorders (Schiller et al., 2008). Therefore, a model system that can show both prefrontal cortex activation and threat-signal and/or safety-signal influences upon the acquisition of avoidance behavior would be advantageous in order to gain a greater understanding of potential sources of anxiety vulnerability.

Male Wistar-Kyoto (WKY) rats exhibit facilitated acquisition of lever-press avoidance when there is a flashing light (FL) presented during the non-shock inter-trial intervals (ITIs); this does not appear to be the case for female WKY rats or Sprague-Dawley (SD) rats of either sex (Beck et al., 2011). Others have documented strain (Powell, 1972; Sutterer et al., 1981; Berger and Starzec, 1988; Overstreet et al., 1990; Escorihuela et al., 1995; Blizard and Adams, 2002; Brush, 2003; Servatius et al., 2008) and sex (Beatty and Beatty, 1970; Gray and Lalljee, 1974; Archer, 1975; Van Oyen et al., 1981; Steenbergen et al., 1990; Heinsbroek et al., 1991; Díaz-Véliz et al., 2000; Beck et al., 2010) differences in avoidance susceptibility; however, WKY rats are a unique rodent, in that, they exhibit qualities of behavioral inhibition (low exploration of novel spaces and stimuli), but they also exhibit rapid acquisition of active-avoidance behavior, which they become resistant to extinguishing (Pare, 1989, 1994a,b, 2000; Servatius et al., 2008; McAuley et al., 2009; Beck et al., 2011; Jiao et al., 2011). This paradoxical combination of behaviorally inhibited temperament and facilitated avoidance acquisition could be due to an added sensitivity to stimuli that predict safety, not just those that predict threat.

The medial prefrontal cortex (mPFC) has a significant role in the acquisition of avoidance behavior in animals (Gabriel and Orona, 1982; Sparenborg and Gabriel, 1990; Shibata, 1993; Kubota et al., 1996; Joel et al., 1997). The infralimbic (IL) cortex region of the mPFC is specifically implicated in the acquisition of two-way shuttle avoidance, by inhibiting the reflexive freezing response that conflicts with running to the safe-side of the apparatus (Moscarello and LeDoux, 2013). Thus, *failure* to exhibit avoidance can be a product of excessive freezing, caused by an overactive central amygdala and/or underactive IL cortex (Choi et al., 2010; Lazaro-Munoz et al., 2010; Martinez et al., 2013). However, the role of prefrontal areas in *increased susceptibility* to acquire active-avoidance behavior (i.e., facilitated avoidance learning) has not been elucidated. Making such determinations is important because avoidance behavior alone is not pathological, but it is the overexpression of avoidance that is pathological.

In a series of six experiments, we sought to determine the role a discrete FL ITI signal has in facilitating active-avoidance learning in WKY rats and the potential neurobiological role of the mPFC in that process. Based on our prior findings (Beck et al., 2011), it appears that the FL ITI-signal facilitates male WKY rat avoidance acquisition in the early phase of training. Hence, our initial experiment was to test whether removing or introducing the ITI-signal mid-acquisition affected overall acquisition in male WKY rats. Next, we conducted two tests of proactive interference to assess the possibility that the FL ITI-signal is perceived as a safety signal. A safety signal in animal behavior is operationalized as a conditioned inhibitor of fear (Rescorla and Lolordo, 1965; Moscovitch and Lolordo, 1968; Rescorla, 1969a); therefore, a safety signal is a stimulus that has acquired certain properties in the animal. If WKY rats perceive the FL ITI-signal as a conditioned inhibitor, then having that same FL serve as a key feature during the training of an unrelated behavior should cause interference (retardation) of the acquisition rate of that newly acquired behavior (Rescorla, 1969b). Similarly, as a conditioned inhibitor of fear reactions, if the ITI-signal is introduced in a separate fear-eliciting situation, its presence should reduce the magnitude of an elicited fear response (summation) (Rescorla, 1969b). Therefore, we examined whether the FL, post-avoidance training, would slow the acquisition of a new conditional response, where a similar FL predicts the occurrence of an unconditional stimulus (CS), using eyeblink conditioning (retardation). This was combined with a parallel experiment to assess whether the avoidance ITI-signal is a conditioned inhibitor of fear using a summation test. Here, we planned to use the avoidance warning signal as an inducer of a fear state using a fear-potentiated startle paradigm, then introduce a FL compound to determine if that state is reduced by the added presence of the light (as a conditioned inhibitor of fear). Following inconclusive results of the retardation/summation tests, we examined whether the ITI-signal served as a feedback signal for the male WKY rats; if so, then shortening the duration of the FL to only the first 5 s of the ITI should be sufficient to facilitate avoidance learning. This was not the case.

As stated above, the inhibition of fear during certain avoidance procedures has been linked to the IL cortex; whereas others have proposed the dorsal prelimbic (PL) cortex increases threat detection. We used these distinctions to try to understand whether IL or PL-cortex serve a role in the acquisition of lever-press active avoidance in male WKY rats, when an ITI-signal is present. First, we examined whether avoidance training with and without an ITI-signal causes differentially expressed neuronal activation (c-Fos expression) across the acquisition process. Again, we hypothesized ITI-induced differential activation of the vmPFC would be most apparent in the early sessions of acquisition. Specifically, the IL cortex of the mPFC should be more activated if there is active processing of “safety,” whereas the more dorsal prelimbic (PL) cortex should be more activated if there is a significant difference in the perception of threat. This was followed by an experiment where either or both the IL and PL cortices were lesioned prior to avoidance training. There was the expectation that IL cortex lesions would slow acquisition of lever-press avoidance responding, if there is an important role for conditioned inhibition of fear; whereas, the PL-cortex lesions would slow acquisition if it is specifically required to perceive threat during the acquisition of lever-press avoidance. Finally, if there needs to be a comparison of safety versus threat in order to acquire the lever-press avoidance behavior, combined lesions may be required to slow acquisition. In sum, these experiments were designed to try to elucidate the function of the ITI-signal in this learning paradigm for the male WKY rats, as well as determine the functional role the mPFC may have in those processes.

## Materials and Methods

### Subjects

Two hundred fifty-six male WKY rats (2–3 months of age upon arrival) were obtained from Harlan Labs (Indianapolis, IN) to serve in one of six possible experiments. Upon arrival, all subjects were maintained on a 12:12 light:dark cycle (lights on 07:00) and had free access to food and water while in the homecages. Room temperatures were maintained in the acceptable ranges as set forth by the NIH Guide for the Care and Use of Animals. Behavioral training occurred at least 14 days post-arrival. In the case of the surgical procedure required prior to eyeblink conditioning (Experiment 2), animals were first trained in lever-press avoidance, and were subjected to the EMG-electrode implantation surgery shortly thereafter (within 1 week of the last session). They were then tested 1 week following the surgery in eyeblink conditioning. All procedures were approved by the VA New Jersey Health Care System Institutional Animal Care and Use Committee, in accordance with *The NIH Guide for the Care and Use of Animals*.

### Avoidance learning

Rats were trained in discrete level-press avoidance behavior for varying durations (ranging from 1 session to 12 sessions, depending on the experiment). In order to accomplish this, Coulbourn Instruments (Allentown, PA, USA) operant chambers, containing a grid floor, a lever, a white light, and a speaker were used in conjunction with Graphic State software. The software controlled the stimulus states in the chamber as well as recorded responses upon the bar within those designated states.

The same parameters previously reported to elicit differences in the acquisition of the avoidance lever-press behavior in WKY rats (Beck et al., 2011) were used for these experiments. Each lever-press avoidance-training session was separated by 1–2 days, with 20 trials conducted per session. For each session, rats were placed in the operant chambers, and, following an initial 60 s non-stimulus period, were exposed to a 60 s warning signal (1 kHz frequency tone at 75 dBA intensity). Following the initial 60 s of the warning signal, intermittent (every 3 s), scrambled shocks (1.0 mA and 0.5 s in duration) were applied to the grid floor. Depressing a lever located on one wall in the test chamber ceased shock presentation (i.e., an escape response). If the lever was depressed in the 60 s period preceding a trial’s first footshock, the shock was avoided. Following each trial, there was a 3 min ITI, when a white cue light, located 10 cm directly above a lever, flashed at a 5 Hz rate (80 lux) for those subjects assigned to the ITI-signal condition. This signal was presented for the entire 3 min ITI, only the first 5 s of the ITI (only Experiment 4), or not at all; however, at no time were shocks administered during the ITI, regardless of the presence/absence of the FL. The opposing wall to the lever was a mounted house-light that provided a baseline low-level of luminance (approximately 40–50 lux), providing enough light for the experimenter to observe the rats when the ITI-signal was not flashing.

### Eyeblink conditioning – retardation test

In Experiment 2, rats were trained in avoidance behavior for 12 sessions prior to the implantation of the necessary electrodes for eyeblink conditioning. Under surgical anesthesia, EMG electrodes were implanted into the orbicularis oculi and associated acrylic-fixed headstages to the surface of the skull (Servatius, 2000). One week following surgery, each rat was first tested for signal quality, while habituating to the test chamber (day 1). For the next 2 days, all rats were exposed to eyeblink conditioning with a 500 ms 82 dB(A) white-noise CS and a 10 ms 10 V eyelid muscle stimulation unconditioned stimulus (US). Every 10 trials comprised a trial-block, which included 1 CS-alone trial, 1 US-alone trial, and 8 CS-US pairings where the CS coterminated with the US (Servatius, 2000; Servatius et al., 2001; Servatius and Beck, 2003). There were 10 trial-blocks per daily session. For the retardation test, we added an 80 lux 5 Hz FL (approximately the same height above the floor as the avoidance chambers) that signaled a US-containing trial. The light flashed for 5 s immediately prior to the CS (on paired trials) and the US on US-alone trials. As such, the FL could be learned as an occasion-setter (OS) for the US (in addition to the acoustic CS) or as a primary CS with a 500 ms trace-interval. We also included a condition where a 1 kHz tone was presented as the OS for 5 s.

### Startle reactivity – summation test

In Experiment 3, rats were exposed to 60 startle test trials, once prior to avoidance training and once following avoidance training (2 days following the last training session). Following avoidance training, it was expected that a tone similar to the avoidance warning signal, preceding the startle pulse, would increase startle reactivity, whereas a co-occurring FL (similar to the ITI signal from avoidance) would reduce that potentiation (Davis and Astrachan, 1978; Hitchcock and Davis, 1987; Grillon et al., 1991). For the startle tests, all rats were given 5 min to acclimate to the testing chamber prior to the initiation of the startle trials. In this test protocol, four trial types were presented in a pseudorandom order, such that no two trial types occurred more than twice within each six trials. The trials were comprised of the following: startle-pulse alone, tone/startle pulse, FL/startle pulse, and tone + FL/startle pulse. Each white-noise startle-pulse stimulus was 100 ms in duration with a 5 ms rise/fall. In the three trial types where there was a preceding stimulus, the preceding stimuli were presented for 5 s. The FL was produced by a similar wall-mounted bulb (as in the avoidance chambers) with 5 Hz flash rate at 80 lux intensity. The 1000 Hz tone, at 75 dB(A) intensity, was produced from two speakers on the ceiling of the startle chamber. The 102 dB(A) startle pulse, produced from the same speakers, followed less than 0.5 s thereafter. The stimulus presentation and data collected from the weight displacement upon the accelerometers (Coulbourn Instruments, Allentown, PA, USA) was conducted through A/D conversion and a custom program written in Labview (National Instruments Corp, Austin, TX, USA). For each startle stimulus presentation, a response threshold for whole body response was computed as the average rectified activity 200 ms prior to stimulus onset plus six times the SD of that rectified activity. Response amplitudes, the maximum rectified activity within 125 ms after stimulus onset, were only recorded when post-stimulus activity exceeded the response threshold. For trials in which activity did not reach this criterion “not available” was recorded, for all others, this calculated value was corrected by each rat’s body weight measured immediately post-testing. These methods for calculating startle reactivity are described in detail elsewhere (Servatius et al., 1994, 1995).

### Immunohistochemistry

In experiment 5, all subjects were randomly assigned to have their brains harvested following a specific training session. Ninety minutes following the assigned session, each rat was prepared for perfusion-fixation via an injection of 150 mg/kg sodium pentobarbital. Once deeply anesthetized, the rats were subjected to transcardial perfusion of 0.9% saline, followed by 10% buffered formalin. Brains were removed, post-fixed in 10% formalin at 4°C overnight, and placed in 30% (weight/volume) sucrose of 0.1 M phosphate buffer solution until the brains sank. A sliding microtome was utilized to slice coronal brain sections of the mPFC (4.20–2.53 mm anterior to Bregma), each with a thickness of 50 μm. All slices were stored in cryoprotectant (0.2 M phosphate buffer solution, glycerin, and ethylene glycol) at −20°C for approximately 4 months. As described elsewhere (Jiao et al., 2011), immunohistochemistry for c-Fos was conducted on every forth mPFC brain section with rabbit anti-c-Fos antibody (1:1000, #sc-52, Santa Cruz Biotechnology, Dallas, TX, USA) for 18 h. Sections were then incubated in biotinylated donkey anti-rabbit secondary antibody (1:200, Jackson ImmunoResearch Laboratories, West Grove, PA, USA) solution for 3 h, followed by incubation in avidin-biotin complex 4°C overnight (Vectastain Standard kit, Vector Laboratories, Burlington, CA, USA). A chromogenic peroxidase oxidation–reduction reaction was performed utilizing nickel-enhanced DAB. Estimates of c-Fos immunoreactive nuclei were obtained using unbiased stereology procedures (Optical fractionator method, Stereo Investigator v. 9.0, MicroBrightField, Colchester, VT, USA). Volume of the ACc, PL cortex, and IL cortex were also obtained to calculate density of c-Fos immunoreactive cells. A Leica microscope with an *x*-, *y*-, and *z*-motorized stage was used. The counting frame had a consistent length-width-height dimension of 80 × 60 × 10. Cell counts and volume regions were counter balanced across hemispheres: half of the rats were counted on the left hemisphere, while the remaining half of the rats were analyzed on the right hemisphere. Counts were performed by an individual blind to the treatment of each analyzed brain. Data are expressed as means of density (cell count/volume) per mPFC region per animal.

### Ventromedial prefrontal cortex lesions

In Experiment 6, male WKY rats were randomly assigned to have lesions to the IL cortex, PL cortex, both PL + IL cortex, or a sham control condition (saline injection). Two to three weeks prior to avoidance acquisition, bilateral lesions to either or both the IL and PL cortex were administered under sodium pentobarbital anesthesia (50 mg/kg, i.p.). The surgical site was prepared and the rat was placed into the stereotaxic apparatus. The coordinates were A/P: +2.9, L: ±1.0 (PL) or 1.5 (IL). Hamilton microsyringes (30 gage needle) were lowered on a 10° angle (PL) or 15° angle (IL). The lowering speed was 0.2 mm/min. Needles were lowered 4.0 mm for PL cortex and 4.5 mm for IL cortex (4.2 mm for combined). Ibotenic acid (5 mg/ml) was delivered in 0.2 μl volumes to single sites and 0.4 μl volumes for the combined PL + IL lesions. All rats recovered under daily administration of banamine and fluids (as necessary).

### Data analysis

Avoidance behavior training in Experiments 1, 4, and 6 was assessed for differences in the emission of lever-press avoidance responses with respect to between-session acquisition and within-session acquisition. Between-session analyses utilized mean session avoidance responses as the dependent measure over sessions, whereas within-session analyses utilized the mean percent of subjects emitting an avoidance response on each trial (collapsed over session blocks of two sessions each) as the dependent measure. A mixed analysis of variance (ANOVA), with session as the repeated measure, was the statistical model used for analysis of the former, and a mixed ANOVA with session block and trial serving as the repeated measures was used for the latter analysis. The between-session analysis provides an overall assessment of avoidance learning over the 4 weeks of acquisition, whereas the within-session analyses provide a means of assessing difference in the acquisition within sessions.

For Experiments 2 and 3 (retardation and summation), mixed designs were also required. A mixed ANOVA, with day and trial block as the repeated measures, was used to assess group differences in the acquisition of conditioned eyeblink responses post-avoidance learning. Similarly, a mixed ANOVA was used to assess group differences in startle magnitude for Experiment 3. All groups experienced all four trial types both pre- and post-avoidance training.

In Experiment 5, the brain analysis required rats to be sacrificed after 1, 2, 4, or 8 sessions of avoidance training. Thus, in order for the avoidance behavior measurement to parallel that of the c-Fos measurements, mean avoidance responses on the day of brain harvest were analyzed via a between subjects ANOVA (no repeated measures). The c-Fos densities across all three regions of the mPFC (IL cortex, PL cortex, and anterior cingulate cortex), were each analyzed via a between subjects ANOVA.

With significant ANOVAs in the above experiments, specific group comparisons were conducted with the Fisher’s LSD multiple comparison test. The probability of making a Type I error was set at 0.05 for all levels of analysis.

## Results

### Experiment 1: Avoidance acquisition with ITI-signal switch

**Figure 1:**
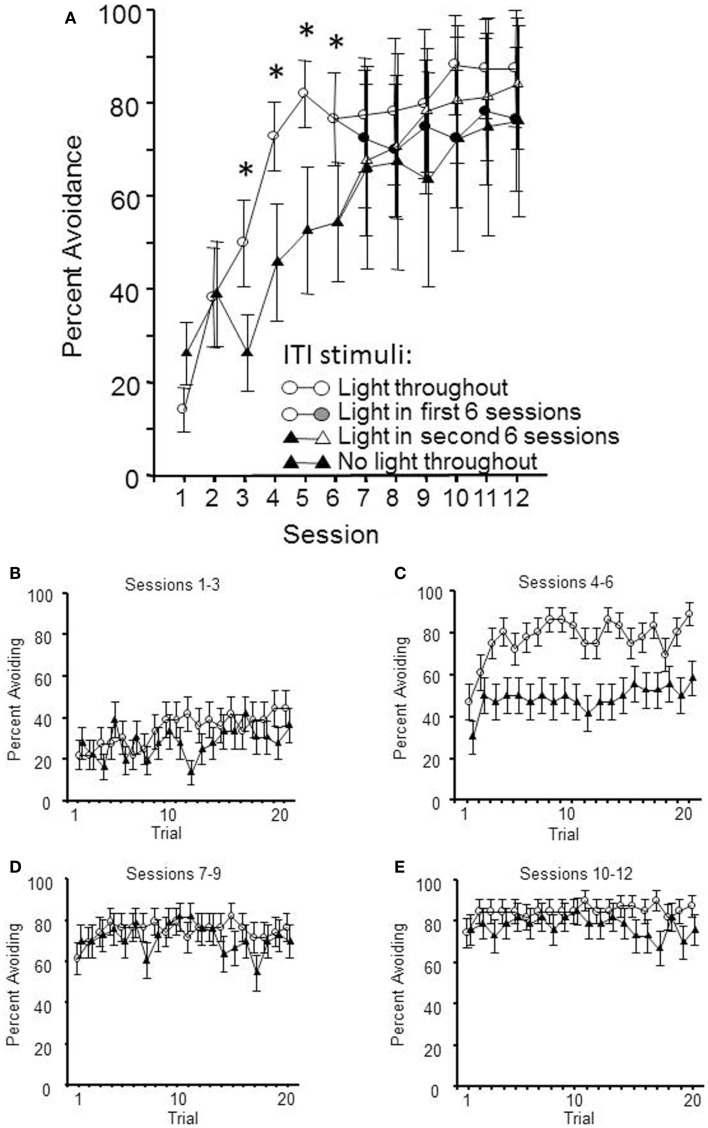
**Lever-press avoidance behavior training occurred over 12 sessions**. At the mid-point of training, following session 6, half of the subjects had their flashing light (FL) ITI-signal status switched. Shown in **(A)** are the mean avoidance responses per condition per session. The facilitation of lever-press avoidance learning by the presence of a FL ITI-signal is evident through session 6, with significant differences between the two initial conditions denoted by an asterisk (*). In the latter half of training, there were no significant differences between the groups, regardless of the presence or absence of the FL during the 3 min ITIs. Shown in **(B,C)** are the percentage of subjects avoiding on each trial through the first (sessions 1–3) and second (sessions 4–6) session blocks, respectively. **(D)** (Sessions 7–9) and **(E)** (sessions 10–12) show the percentage of subjects avoiding after half of each group had an ITI-signal switch. This within-session analysis demonstrates that the presence/absence of the FL during the ITIs does not affect the between-session retention of the learning, as much as the within-session acquisition process.

Thirty-two male WKY rats were randomly assigned to begin lever-press avoidance training with or without a FL ITI-signal for half of acquisition (Initial Condition, sessions 1–6); half of each group subsequently had that signal present or absent for sessions 7–12, thus creating four distinct groups (final condition). The hypothesis was that a safety signal would enhance the acquisition rate of the avoidant behavior. As shown in Figure 1, the expected difference in acquisition between those training with and without the ITI signal was replicated in the first half of training; WKY rats acquire quicker when there is a FL presented during the ITIs. This impression was confirmed by a significant main effect of session, *F*(5, 110) = 20.7, *p* < 0.001 and a significant group × session interaction, *F*(5, 110) = 4.3, *p* < 0.001. However, through the second half of acquisition sessions, the group differences ceased to exist. To this end, only a main effect of session was calculated, *F*(5, 100) = 5.0, *p* < 0.001. Additional analyses were conducted on the number of non-reinforced responses emitted during each min of the ITI. These failed to detect any difference in the amount of responding during the ITI that was attributable to the presence/absence of the FL during the ITI.

Within-session acquisition was assessed through two repeated measures ANOVAs, one analyzing the first half of training (shown in Figures 1B,C) and a second analyzing the second half of training (shown in Figures 1D,E). The first half of acquisition was analyzed using a 2 (initial condition) × 2 (session block) × 20 (trial) ANOVA. Each three consecutive sessions comprised a session block. This analysis yielded significant main effects of session block, *F*(1, 22) = 41.2, *p* < 0.001 and trial, *F*(19, 418) = 4.2, *p* < 0.001, complemented by an initial condition × session block interaction, *F*(1, 22) = 5.0, *p* < 0.03. The *post hoc* analyses found that these significant effects recapitulate the between-session analyses showing acquisition over sessions (with the ITI-signal group acquiring faster), and there is the added confirmation of within-session learning as well. However, there was no interaction between within-session learning (trial) and initial condition. The second phase of training was analyzed via a 2 (initial condition) × 2 (final condition) × 4 (session block) × 20 (trial) ANOVA. These analyzes yielded significant main effects of session block, *F*(1, 20) = 8.1, *p* < 0.01 and trial, *F*(19, 380) = 1.8, *p* < 0.02, with an additional significant final condition × trial interaction, *F*(19, 80) = 1.7, *p* < 0.03. *Post hoc* analyses found that the groups with the ITI-signal in sessions 7–12 exhibited more avoidance responses in the later trials of those sessions (trials > 13). Thus, differences in within-session learning were evident across acquisition, with the differences due to the presence/absence of the ITI-signal being predominately reflected in the latter trials within those sessions.

The results of this experiment suggest that the greatest effect the ITI-signal has on acquisition of avoidance behavior is during those first few sessions after the transition from mostly escape responding to predominantly avoidance responding. Moreover, any ITI-signal associated differences in acquisition, within sessions, are reflected through differences in attaining asymptotic response levels.

### Experiment 2: ITI-signal proactive interference test – retardation

Sixty-four male WKY rats were initially trained for 12 sessions of lever-press avoidance (data not shown). Seven rats were removed due to a lack of acquiring the avoidance behavior. The remaining 57 were subsequently trained in eyeblink conditioning to determine if the presentation of stimuli experienced in avoidance training would cause proactive interference for the acquisition of conditioned eyeblink responses (a retardation effect). Either the warning signal (tone) or the ITI signal (FL) from the avoidance training was presented 5 s prior to the introduction of conditional stimuli (CSs) and/or unconditional stimuli (USs). The CS-preceding stimulus (either tone or FL) was more predictive of the US than the CS (due to the fact there are CS-alone trials and US-alone trials). The expectation was that the presentation of the warning signal or ITI signal as an occasion setter for the US would slow eyeblink conditioning (i.e., retardation through proactive interference) because the stimulus will have already been associated with conditions from avoidance learning. A control group with no additional OS stimulus presentation was included to discern if the novel experience of experiencing the FL during eyeblink conditioning, as an occasion setter for the US, would facilitate acquisition above that of those without any occasion setter (the normal control condition). Of the 57 WKY rats trained in eyeblink conditioning, five additional rats were removed following analysis of the signal (poor signal quality).

**Figure 2:**
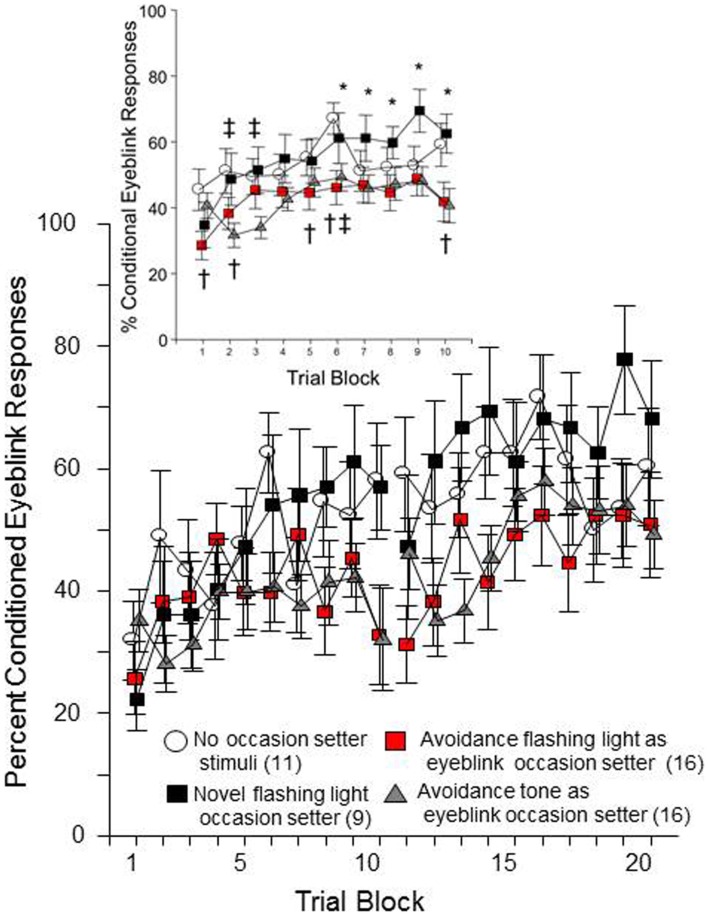
**Male WKY rats, previously trained in lever-press avoidance, were conditioned to reflexively blink to a white-noise conditional stimulus (CS), which was paired with unconditional eyelid muscle stimulation (US)**. Prior to the CS on trials where the US was to be presented, either a 5 Hz flashing light (FL) or a 1 kHz tone was presented as an occasion setter for the US. Those rats trained with the FL during avoidance, then subsequently trained to emit conditioned eyeblink responses with a FL occasion setter during eyeblink conditioning (denoted in red), acquired conditional eyeblink responses slower than those with an occasion-setter FL that previously were trained in avoidance but did not have a FL during the avoidance ITIs (i.e., novel FL). Rats trained with an occasion setter that approximated the warning tone from the previous avoidance learning were also slower to acquire the response compared to the novel FL occasion-setter group. Significant differences between groups were found across trial block (collapsed over day), see insert. An asterisk (*) represents a significant difference between the two conditions with a FL occasion setter. A cross (†) represents a significant difference between the no-occasion-setter control group and the avoidance ITI-signal/FL occasion-setter group. A double cross (‡) represents a significant difference between the no-occasion-setter control group and the tone occasion-setter group (*p* < 0.05 Fishers LSD).

As shown in Figure 2, all groups of rats emitted more conditioned responses over each session of both conditioning days. This impression was confirmed by significant main effects of day, *F*(1, 48) = 31.4, *p* < 0.001 and trial block, *F*(9, 432) = 9.3, *p* < 0.001. Still, it is clear that the four groups differed in their rate of conditioned response expression over training. This impression was confirmed by a significant group × trial-block interaction, *F*(27, 432) = 1.7, *p* < 0.02. *Post hoc* analyses confirmed that there are specific group differences in the acquisition of eyeblink conditioned responses over trial-blocks each day. As predicted, the groups that had a FL occasion setter differed based on prior experience with the FL during avoidance training. Those that had previously experienced the FL, as an ITI signal in avoidance training, emitted significantly fewer conditioned eyeblinks in trial blocks 6, 7, 8, 9, and 10 than the group for whom the FL was a novel stimulus. Moreover, those rats with experience of the FL during avoidance emitted fewer conditioned responses, compared to the no-OS control group on trial blocks 1, 2, 5, 6, and 10. The tone occasion setter also caused fewer conditioned eyeblinks, comparing the tone OS group to the no-occasion-setter group in trial blocks 2, 3, and 6. Thus, although the 5 s FL occasion setter did not appreciably facilitate learning of the conditioned response above that of the no-occasion-setter condition, rats that had prior experience with the FL were less likely to emit conditioned responses. This suggests acquisition of a second learned response to the ITI-signal, as well as the warning signal, is mildly retarded due to the prior exposure to those stimuli during avoidance learning (i.e., proactive interference).

### Experiment 3: ITI-signal proactive interference test – summation

Sixteen male WKY rats were matched on their baseline startle magnitudes on pulse-alone trials, and randomly assigned to be trained in lever-press avoidance with a FL ITI-signal or not. The day following the 12th and final avoidance-training session, all rats were re-tested for startle reactivity. As with the pretest, there were four trial types: pulse alone, 5 s tone/pulse, 5 s FL/pulse, and 5 s FL + tone/pulse. Assuming the avoidance protocol, warning tone (post-acquisition) would enhance startle reactivity (i.e., fear-potentiated startle), the FL was expected to dampen that enhancement if the FL acquires the properties of a safety signal (for those rats trained with the FL as the ITI-signal).

**Figure 3:**
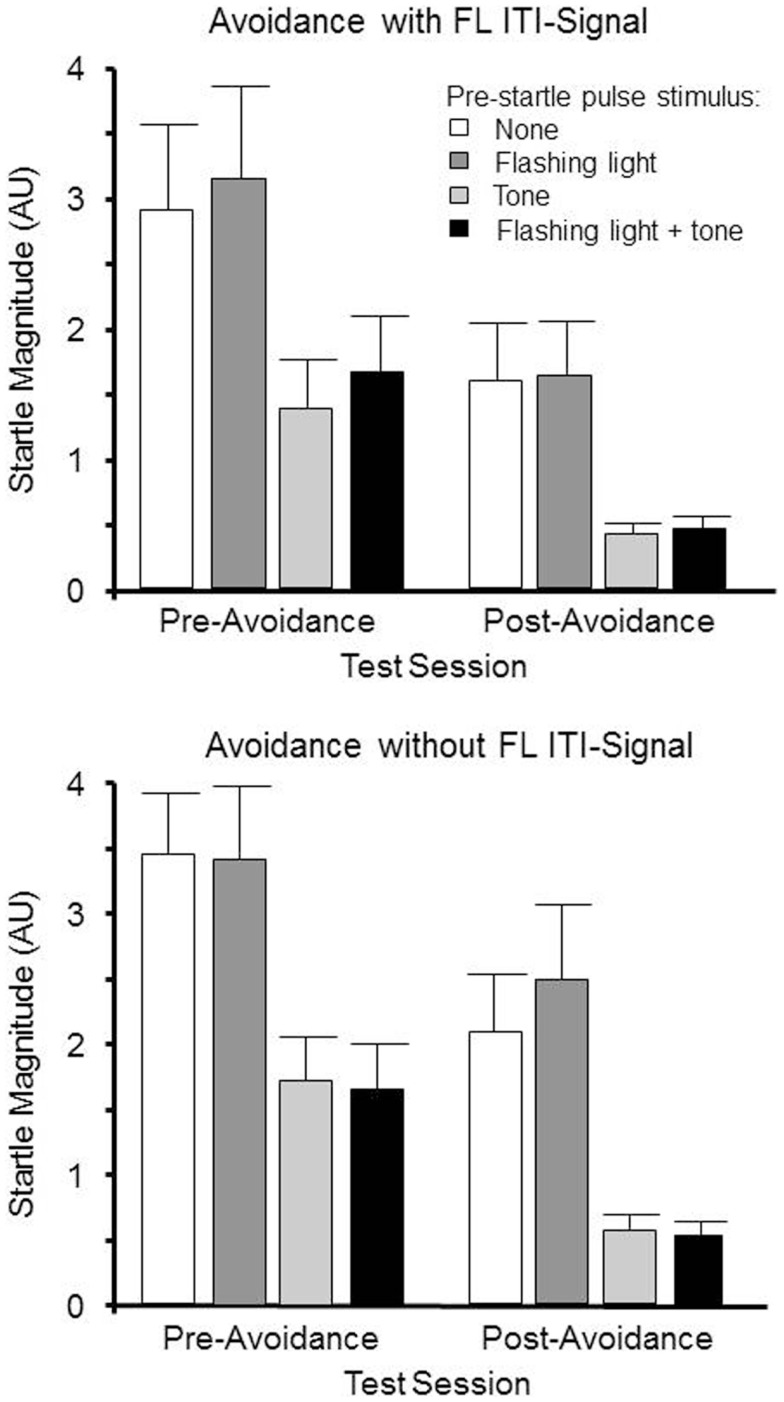
**Male WKY rats were pretested for startle reactivity prior to avoidance training**. Of the 24 startle trials, 8 were preceded by a 5 s 1 kHz tone, 8 were preceded by a 5 Hz FL, 8 were preceded by the combination of both the tone and FL, and 8 were not preceded by any stimuli. The same startle test occurred within 2 days following the end of lever-press avoidance training. The elicited startle responses were lower during the post-avoidance test, regardless of begin trained with or without a FL ITI signal. However, exposure to the 1 kHz tone reduced startle magnitudes approximately 50–60%. The FL did not appear to influence the magnitude of the elicited startle response.

One rat from the ITI-signal-trained group was removed from the study, as it did not meet the requirements of having acquired the avoidance behavior, leaving seven ITI-signal-trained and eight non-ITI-signal-trained rats to be analyzed across both pre and post-avoidance startle tests to determine if the acquired properties of the tone and FL during avoidance learning can potentiate or dampen the subsequently elicited startle reflex. The resulting 2 (group) × 2 (session) × 4 (trial type) mixed ANOVA failed to detect any differences due to being trained in avoidance with or without the FL ITI-signal. Only main effects of session, *F*(1, 13) ( 18.9, p ( <.0010.001 and trial type, F (3, 39) ( 47.5, p ( <.0010.001 were evident. As evidenced in Figure 3, post-avoidance startle tests had significantly lower startle magnitudes, independent of avoidance-training ITI-signal group assignment. Moreover, preceding startle pulses with an equivalent tone as the avoidance warning signal reduced the magnitudes of the elicited startle responses. Unexpectedly, this was even the case prior to the avoidance training. Subsequent avoidance training with the tone was a warning signal did not change this pattern. These findings suggest the tone had startle dampening properties that 12 avoidance acquisition sessions are not sufficient to overcome, through eliciting a fear or anxiety-potentiated startle response prior to the startle pulse. The FLflashing light alone had no discernible effect on the elicited startle response. Thus, for those rats trained with the FLflashing light ITI-signal, subsequent exposure to the FLflashing light prior to startle pulses does not reduce the vigilance of the rats.

### Experiment 4: Shortened ITI-signal

**Figure 4:**
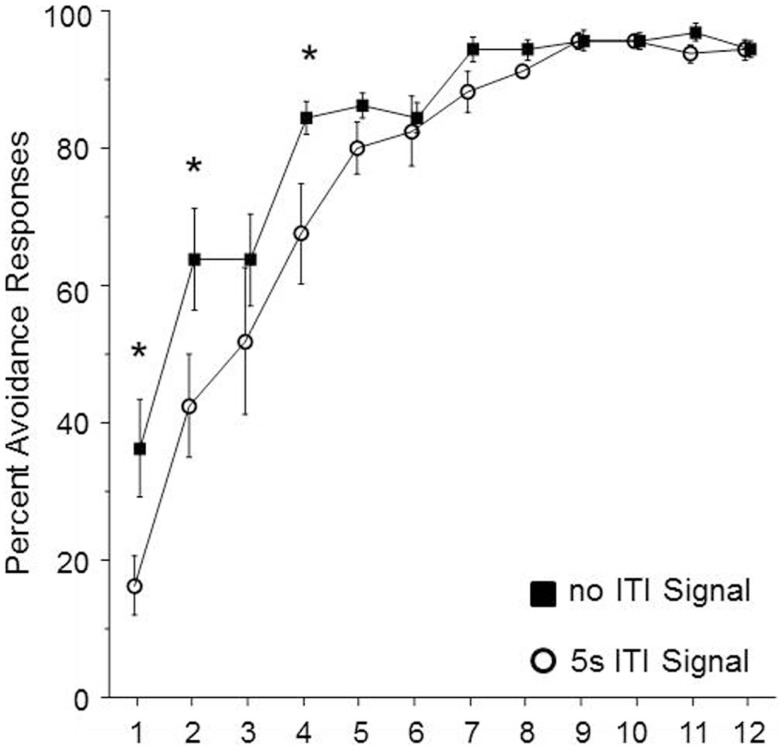
**Male WKY rats were randomly assigned to be trained in lever-press avoidance with no ITI-signal or a 5 s flashing light (FL) ITI-signal (in both cases, the total ITI was 3 min)**. Those assigned to the 5 s FL condition expressed significantly less avoidance responses over the first four sessions of acquisition. An asterisk (*) represents a significant difference between groups for a particular session (*p* < 0.05, Fishers LSD).

In order to determine whether the duration of the ITI-signal is a critical element for the facilitation of avoidance acquisition in male WKY rats, the duration of the FL was shortened to the first 5 s of the 3 min ITI. Sixteen male WKY rats were randomly assigned to be trained with either a shortened signal or no signal during the 3 min ITI. As shown in Figure 4, the groups differed in performance over the first four sessions of acquisition. This impression was confirmed by significant main effects of group, *F*(1, 14) = 5.7, *p* < 0.05 and session, *F*(11, 134) = 60.2, *p* < 0.001, as well as a significant group × session interaction, *F*(11, 154) = 2.0, *p* < 0.05. *Post hoc* analyses confirmed that the two groups differed in the percentage of avoidance responses emitted during sessions 1, 2, and 4, with the no-signal group emitting more avoidance responses during those three sessions.

### Experiment 5: Prefrontal activation during avoidance acquisition

Based upon past findings with fear conditioning and lever-press avoidance, the mPFC cortex was expected to exhibit sub-region specific responding during the acquisition process and based on the presence/absence of a FL ITI-signal. The anterior cingulate cortex was expected to exhibit greater activation once avoidance was acquired. The IL cortex was predicted to exhibit greater neuronal activation following the perception of perceived safety, whereas the PL cortex was predicted to exhibit greater activation following the perception of perceived threat. Thus, greater activation in IL cortex would support the theory that the ITI-signal is being processed as a safety signal, but greater PL-cortex release would suggest greater perceived threat.

**Figure 5:**
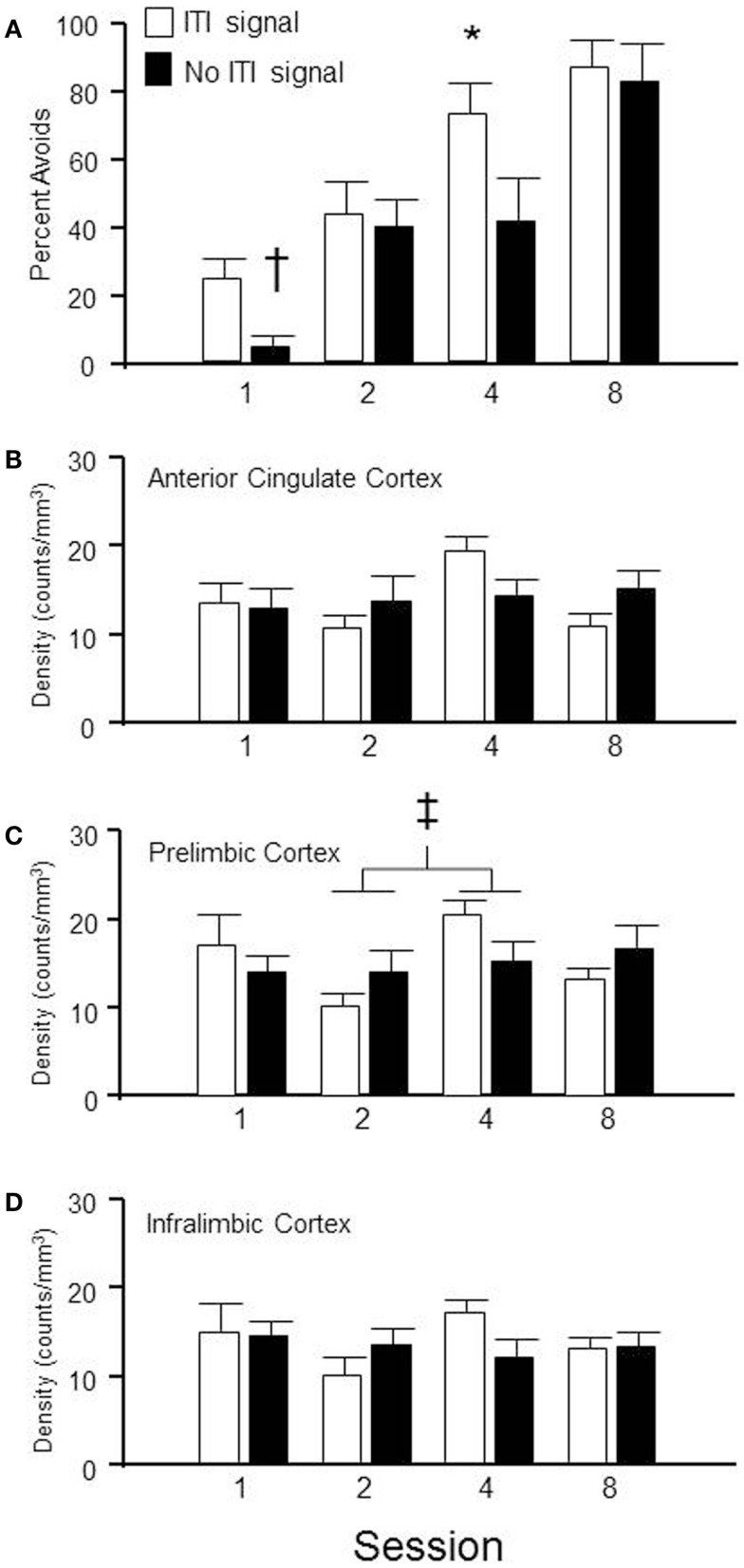
**Brains were extracted from subjects, trained in the lever-press avoidance protocol with either the FL ITI-signal or no explicit ITI-signal (nFL), following a randomly assigned number of training sessions**. Shown in **(A)** is the percentage of avoidance responses emitted from the rats on the day they were sacrificed. **(B–D)** provide the density of c-Fos labeling in the anterior cingulate (AC) cortex, prelimbic (PL) cortex, and infralimbic (IL) cortex, respectively, for those subjects depicted in **(A)**. Behaviorally, the groups with a flashing light (FL) ITI-signal emitted more lever-press avoidance responses during sessions 1(†) and 4 (*) than the groups without an ITI-signal. Neurochemically, only the PL cortex exhibited significant differences between groups. Overall, the density of c-Fos labeling was significantly different between sessions 2 and 4, regardless of the ITI signal (‡).

Sixty-four male WKY rats were randomly selected to be sacrificed at different stages of lever-press avoidance acquisition, with the goal of determining the subregions of the mPFC that are most activated at each stage and whether the presence of the ITI-signal influences that neuronal activation. As shown in Figure 5A, acquisition of the lever-press escape-avoidance behavior occurred in both the ITI-signal and non-ITI-signal groups. These data were analyzed via a 2 (group) × 4 (session) between subjects ANOVA, which calculated significant main effects of group, *F*(1, 54) = 5.5, *p* < 0.02, and session, *F*(3, 54) = 20.6, *p* < 0.001. *Post hoc* tests suggested the ITI-signal/no-signal groups differed from each other prior to session 4, with the non-signal group exhibiting less avoidance behavior. The general pattern of the ITI-signal groups exhibiting faster acquisition early in training was replicated. Further, significant differences in ITI-responding were assessed via a 2 (group) × 4 (session) between subjects ANOVA. These analyses detected a significant main effect of session, *F*(3, 50) = 5.9, *p* < 0.002, but no effect of group. Overall, more non-reinforced ITI responses were emitted during the second session compared to all other sessions, regardless of signal condition (data not shown).

Group × session (2 × 4) between subjects analysis of variance was utilized to determine regional differences in c-Fos immunoreactivity density in anterior cingulate cortex, PL cortex, and IL cortex. The only significant difference was found in the c-Fos density in PL cortex (see Figure 5C). A main effect of session was found, *F*(3, 49) = 2.7, *p* < 0.05. Session 2 c-Fos densities were significantly different than session 4 c-Fos densities, regardless of group assignment. No significant differences were detected in anterior cingulate cortex (Figure 5B) or IL cortex (Figure 5D). This suggests mPFC is involved in the process of acquisition but its level of activation is not appreciably influenced by the presence/absence of an explicit ITI-signal, even when that ITI-signal appears to facilitate the transition from escape to avoidance responding.

### Experiment 6: Ventromedial prefrontal cortex lesion effects on avoidance

**Figure 6:**
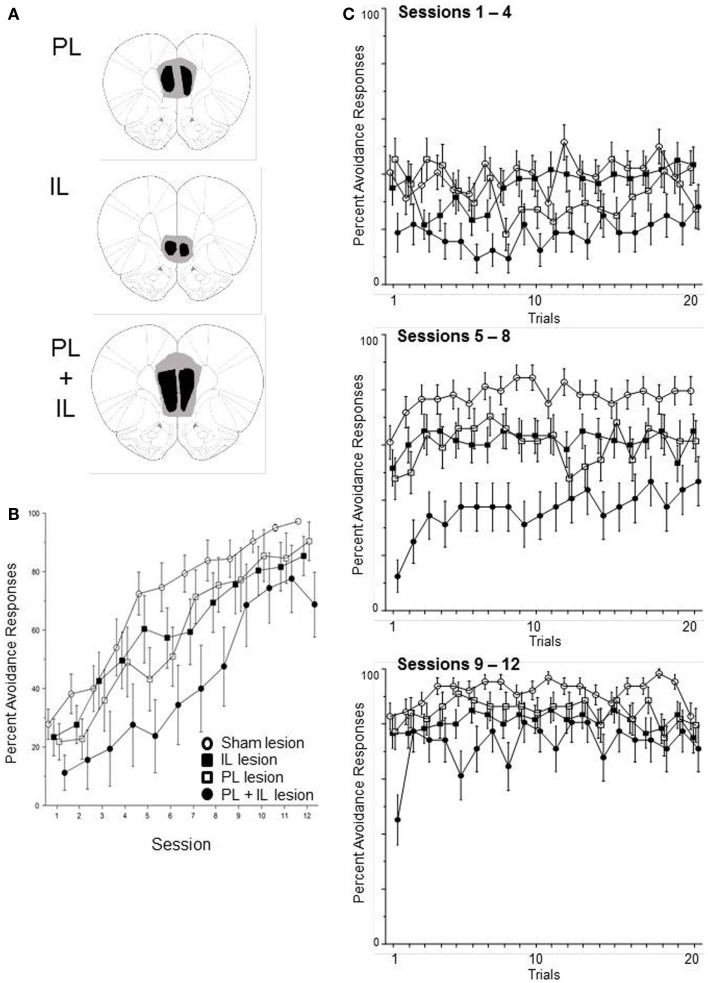
**Male WKY rats were randomly assigned to have bilateral excitotoxic lesions to the PL cortex, IL cortex, or combined PL + IL cortex [see (A)], followed by lever-press avoidance training, beginning at least 10 days later**. As shown in **(B)**, the lesion groups did not significantly differ from the sham-lesion group when assessed across sessions (until the PL + IL group was specifically compared to the Sham group). As shown in **(C)**, within-session acquisition of the response did not suggest the lesion altered the characteristic phenotype of WKY rat lever-press avoidance, that is the absence of a warm-up effect (the seemingly reacquisition of the response beginning at a performance level lower than what the rats had attained in the previous session). Even the PL + IL lesion group, which exhibited the slowest avoidance rates, did not exhibit a warm-up effect. Thus, these lesions did not reinstate a typical warm-up pattern of responding.

Sixty-four male WKY rats were subjected to excitotoxic lesions to the PL, IL, or combined PL and IL cortex. Confirmation of target site damage (see Figure 6A) required one IL, three PL, and eight PL + IL lesion rats to be dropped due to failure of bilateral lesions in both targets. In addition, two PL-lesion rats were dropped due to failing to acquire an escape response within five sessions. This yielded the following groups: sham (16), IL (15), PL (11), and PL + IL (8). The percentage of avoidance responses emitted of those rats were analyzed via a 4 (group) × 12 (session) mixed ANOVA. The complete analysis only produced a main effect of session, *F*(1, 11) = 60.8, *p* < 0.001. As observed in Figure 6B, all four groups exhibited a significant increase in lever-press avoidance responding over the 12 acquisition sessions. Hence, it is clear that neither the PL or IL cortex is necessary for the acquisition of lever-press avoidance behavior in male WKY rats. Still, there appears to be a general trend for larger lesions (PL + IL) to somewhat slow the acquisition process. Given this observation, we removed the PL cortex-alone and IL cortex-alone groups from the analysis to assess whether a vmPFC lesion (PL + IL cortex) significantly slows acquisition of lever-press avoidance behavior. This analysis yielded significant mains effect of group, *F*(1, 22) = 8.7, *p* < 0.01 and session, *F*(11, 241) = 28.3, *p* < 0.001. These results suggest more extensive bilateral damage to the vmPFC slows acquisition, but avoidance acquisition is still quite significant over sessions. Thus, the vmPFC is not necessary for lever-press avoidance in male WKY rats, but its actions may contribute to the normally rapid acquisition and higher asymptotic performance levels normally displayed in male WKY rats.

As above, we also conducted within-session analyses to determine if the lesions affected one of the most prominent features of lever-press avoidance learning in WKY rats – the absence of avoidance warm-up. Acquisition was separated into three phases (session blocks) for the within-session analyses. Thus, a 4 (group) × 3 (session block) × 20 (trial) mixed ANOVA determined differences in the percentage of subjects that emitted avoidance responses on specific trials within the session blocks (early acquisition, mid-acquisition, and late acquisition). As evidenced in Figure 6C, the session differences in Figure 6B predominately reflect differences in within-session acquisition. This impression is supported by a significant main effects of session block, *F*(2, 92) = 116.1, *p* < 0.001 and trial, *F*(19, 874) = 2.8, *p* < 0.001, as well as a significant session block × trial interaction, *F*(38, 1748) = 2.5, *p* < 0.001. Despite the obvious difference in the PL + IL lesion condition, a main effect of group and a group × trial interaction failed to attain significance (*p* = 0.09 and *p* = 0.08, respectively). Across-session analyses for detecting early trial warm-up versus first-trial avoidance failed to detect any significant differences that would be indicative of warm-up at the beginning of either session block 2 or 3.

A within subjects analysis also was conducted on the emitting of non-reinforced lever-presses, during each of the 3 min of the ITI, to determine whether lesions of the mPFC influence the emitting of those responses. Thus, a 4 (group) × 3 (session block) × 3 (ITI Min) mixed ANOVA was used for determining group differences across acquisition, as well as, across the 3 min of the ITI. There was only a significant main effect of ITI Min, *F*(2, 92) = 265.6, *p* < 0.001. Over acquisition, the mean number of lever-presses emitted in each of the ITI minutes was 2.0 ± 0.05, 0.7 ± 0.03, and 0.6 ± 0.03, respectively. There were no significant effects of group or session block (all *p*’s > 0.08).

**Figure 7:**
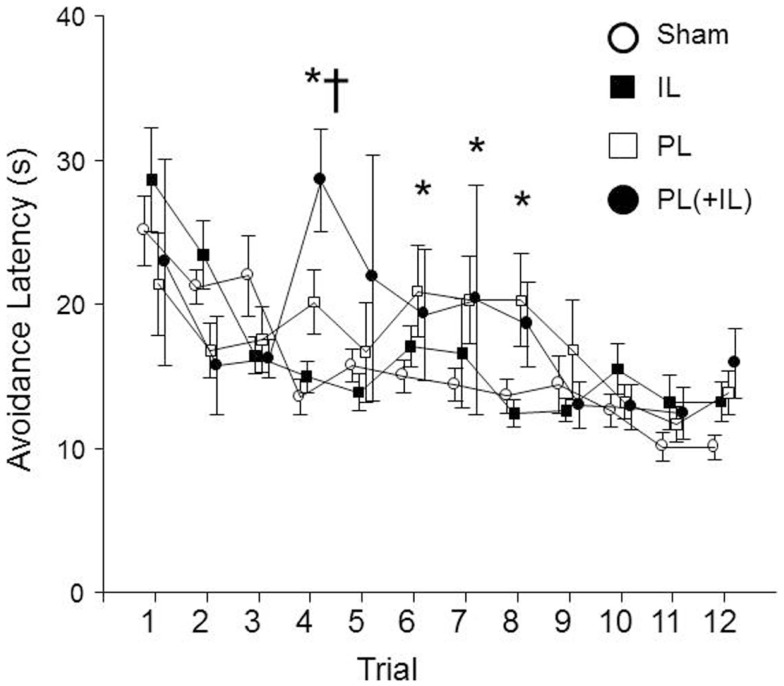
**The change in avoidance response latency was affected by the lesion in male WKY rats**. As denoted by the asterisk (*), the PL-cortex lesion group has slower avoidance latencies beginning with session 4, the session where equal or more avoidance responses are typically emitted (Fisher LSD, *p* < 0.05). Thus, the shorter latencies prior to session 4 are due to much fewer avoidance responses being represented in the calculation, then the more representative mean values for the latencies are longer through session 8. The cross (†) represents the combined PL + IL lesion group being significantly different from the Sham-lesion condition during session 4 only.

Finally, we assessed whether the lesions may have affected the timing of the emitting of the avoidance responses. A group × session mixed ANOVA determined that there was the expected main effect of session, *F*(11, 396) = 9.4, *p* < 0.001, as latencies of the avoidance responses decreased over sessions (see Figure 7). However, an additional significant group × session interaction, *F*(33, 396) = 2.0, *p* < 0.005 suggested that there was a differential decrease among the lesion groups. The *post hoc* analyses found the combined PL + IL lesion group was significantly different from the Sham-lesion group for the fourth session, but the PL-lesion group was significantly different from the Sham-lesion group for sessions 4, 6, 7, and 8. The IL-group was never significantly different from the Sham-lesion group.

## Discussion

The use of explicit ITI signals has occurred in active-avoidance learning for over 40 years (Dillow et al., 1972; Berger and Brush, 1975; Berger and Starzec, 1988; Brennan et al., 2003); yet, rarely has the actual neurobehavioral role of extra stimuli been specifically studied. Previously, we established that male WKY rats acquire lever-press active-avoidance behavior quicker than male SD rats, and that this facilitation was completely attributable to the presence/absence of a FL during the 3-min ITIs that followed each trial (Beck et al., 2011). Here, we established that the FL ITI-signal appears to acquire some mnemonic properties in the male WKY rats, as evidenced by the mild proactive interference it causes when it is introduced into another learning paradigm following avoidance training (i.e., a mild retardation effect on eyeblink conditioning). However, the enhancing effect of the ITI-signal has a temporal component. When the duration of the signal was reduced to the first 5 s of the ITI, male WKY rats were slower to acquire lever-press avoidance compared to their non-signaled counterparts. This temporal element to the ITI-signal could be viewed as support for its role as a safety signal, but higher c-Fos activation of the IL cortex would be expected for a conditioned inhibitor of fear, which was not observed. Instead, a significant change in c-Fos activation was observed in the PL cortex between session 2 and 4, the period where the rats transition from escape responding to avoidance responding. The first 4–6 sessions are also the period where the presence of the FL during the ITI could facilitate or slow acquisition (depending on duration of exposure). Yet, lesions to either the PL or IL cortex did not appreciably slow acquisition of the lever-press avoidance behavior, but lesions to both regions did cause slower acquisition compared to sham controls. One interpretation of this additive effect is that either a cortical evaluation that inhibits fear (IL) or enhances perceived threat (PL) is capable to support rapid acquisition in male WKY rats, but when neither cortical evaluation is functional, the acquisition is slowed (although still clearly evident). In all, these data suggest the mPFC can modulate the acquisition of lever-press avoidance in male WKY rats, and a FL, which lasts the duration of the ITI, acquires associative properties enhances avoidance acquisition; yet the mPFC neuronal activation and the critical period for its effectiveness do not necessarily support its role as a conditioned inhibitor or fear (i.e., safety signal).

The IL cortex, not PL cortex, has been shown to be positioned in the cortico-limbic network of emotional/motivational responsiveness as a primary inhibitor of amygdala-dependent fear reactions (e.g., conditioned freezing) (Quirk et al., 2006). Recent work has expanded the role of the IL cortex to a necessary structure for shuttle-avoidance learning, in that, conditioning to the warning signal can cause the rats to freeze, thus impeding their ability to run to the “safe” chamber (Moscarello and LeDoux, 2013). This finding complements those that have shown poor shuttle-avoidance learners can be “saved” by causing a lesion to the central amygdala, which releases the rats from emitting species-specific conditioned freezing responses (Choi et al., 2010). However, this competing freezing effect may be paradigm specific. For example, inactivation of IL cortex increases freezing in a step-up avoidance paradigm, but it does not to the extent that it impairs emitting the avoidance behavior (Bravo-Rivera et al., 2014). Still, IL cortex may be required for the extinction-learning that instills a lasting reduction in avoidance behavior (Bravo-Rivera et al., 2014). This was also recently observed in a fear/safety versus reward discrimination paradigm. In that paradigm, IL cortex inactivation did not affect the reduction of freezing to a compound fear-inducing CS with a safety signal; however, IL cortex inactivation increased freezing to both the CS and compound CS/safety signal during extinction recall (Sangha et al., 2014). In the current study, we did not observe any difference in IL c-Fos activation throughout acquisition of lever-press active-avoidance behavior, and lesions to the IL cortex did not appreciably affect acquisition of lever-press avoidance. The lack of difference over sessions and between ITI-signal/no ITI-signal male WKY rats suggest any role the IL cortex has in lever-press avoidance is not particularly sensitive to changes in stimulus perception or motor responding that occur over acquisition sessions. This further supports the growing literature suggesting the role for IL cortex in avoidance behavior may be paradigm specific. If a motor response needs to be inhibited (e.g., freezing) then IL cortex is quite important; however, if the paradigm is less sensitive to conflicting reflexive fear responses, then the IL cortex may not be required. Thus, the role of IL cortex may be specific to the processes involved in the acquisition of motor inhibition, rather than serving a role of acquiring associations linked to conditions of perceived safety (versus threat).

Despite the fact that the IL cortex may not be involved in the processing of perceived safety, the FL during the ITI-signal may still be processed as a safety signal through other brain regions. Tests of retardation and summation were conducted to test whether the ITI-signal is perceived as a safety signal. Introducing a similar FL in another associative learning paradigm, following avoidance acquisition with a FL ITI signal, caused proactive interference of the newly acquired reflexive conditional eyeblink response. As a novel stimulus, the FL did not appreciably influence the acquisition of eyeblink CRs; therefore, prior experience with the FL did cause interference whereas it normally would not affect the acquisition. This apparent demonstration of proactive interference is important for two distinct reasons. First, it confirms the FL, as an external stimulus, likely acquires associations to some aspects of the avoidance learning situation. Even though habituation to the FL may appear to be a parsimonious explanation for both the lack of difference over time in avoidance acquisition and no additional acquisition-enhancing effect in eyeblink conditioning, we would have expected dishabituation to the FL in the novel eyeblink conditioning test chambers, as habituation to repeated external stimuli, during emotional learning, is generally context dependent (Hermitte et al., 1999; Tomsic et al., 2009). Second, previous work suggests abnormal proactive interference in WKY rats. Specifically, latent inhibition to an auditory CS, used subsequently in eyeblink conditioning, could be elicited in SD rats following 30 pre-exposures; WKY rats were unaffected by the same amount of pre-exposure (Ricart et al., 2011a). In contrast, in the current study, we appear to have proactive interference in male WKY rats. Granted, although the FL was positioned as an added CS in eyeblink conditioning, we cannot conclude that the rats specifically utilized the FL signal in combination with or instead of the auditory CS. Moreover, the group with the novel FL OS did not differ from those trained without an OS. Previously, we observed a similar non-significant trend toward facilitation of eyeblink conditioning in WKY when a 5 Hz light was flashed throughout the entire session (Beck et al., 2011). In contrast to male SD rats, which exhibited a significant facilitation of acquisition with the constant FL, the effects in WKY rats suggest the additional stimulus may only be providing a relatively mild increase in arousal or attention to the subsequent CS. Interestingly, since the re-introduction of the avoidance tone as an OS for eyeblink conditioning had a comparable effect as the re-introduction of the FL, we can conclude that any retardation effect is not specific to an association formed to the ITI signal. Further, this suggests the proactive interference observed pertains to all the signals acquired during the avoidance training, not just the signal of “safety.” Therefore, the specificity of the retardation of learning cannot be attributed to the ITI-signal specifically serving as a safety signal.

Next, we tested whether the ITI-signal could pass the criterion of summation. Following a similar design as human tests of safety-signal inhibition of fear-potentiated startle (Grillon et al., 1994), we unexpectedly found the mere exposure to a 1-kHz tone prior to a startle pulse was sufficient to significantly dampen the magnitude of the motor response. Historically, rodent fear-potentiated startle paradigms have utilized either a light or a sound as the CS. For those studies that have utilized an acoustic CS, 3–5 s is a common CS duration (Brown et al., 1951; Kurtz and Siegel, 1966; Hitchcock and Davis, 1987; Fendt et al., 2005). In order to best match the warning signal, we had the tone produced at an intensity of 75 dB(A). This intensity is higher than that used in some studies (Kurtz and Siegel, 1966) but not others (Fendt et al., 2005). We considered that this dampening of startle reactivity may have been due to the use of male WKY rats, as other have reported poor fear conditioning in this strain (Pardon et al., 2002), but we tested this startle protocol with male SD rats and obtained similar pre-avoidance results (unpublished observation). It is a common procedure to habituate the startle reflex prior to the assessment of startle potentiation (Rosen et al., 1996); however, in this case, the tone CS suppressed the startle response more than any within-session habituation to the pulse alone (50% of the pulse-alone trial magnitudes). Moreover, male WKY rats have been shown to exhibit higher startle magnitudes than male SD rats (Ricart et al., 2011b); hence, even extended habituation may not have equated pulse-alone trials to those with the preceding tone. Further research will need to determine what factors led to this substantial suppression of the startle response by the tone that was to serve as the CS, but, nonetheless, after the surprising results of the pretest, there was the possibility that the tone would acquire aversive properties during avoidance training. This was clearly not the case. It instead confirms previous experimentation, with other endpoints, which were interpreted to reflect a decrease in elicited fear to the CS in avoidance paradigms (Starr and Mineka, 1977). Any future test of summation with respect to the FL ITI signal will undoubtedly require a distinct aversive learning situation from that of the avoidance paradigm, thereby removing the additional issues of using the acoustic warning signal and any reduction in fear to that signal that develops over training.

With respect to the ITI-signal, the data derived from these experiments do not support the role of the ITI-signal as a conditioned inhibitor of fear. Although we could argue a summation or retardation test following the initial 1 or 2 sessions of escape-avoidance training may provide stronger evidence that the FL ITI-signal acquires the properties of a conditioned inhibitor, i.e., safety signal, the IL cortex is positioned to be a likely neuronal source of inhibition upon the amygdala (Quirk et al., 2006). The lack of differential IL cortex activation throughout training and the lack of IL cortex lesions on avoidance acquisition suggests the presence or absence of the FL during the ITIs is not activating an inhibitory response upon the amygdala via the IL cortex. Granted, other areas of the brain have also been suggested to process aspects of “learned safety” outside of the vmPFC and the amygdala, such as the insular cortex and bed nucleus of the stria terminalis (Christianson et al., 2008, 2011), but those circuits have been specifically implicated in coping with uncontrollable stressors, which is not the case in this avoidance paradigm.

Differences in neuronal activation were evident from session 2 to session 4 in the PL cortex. This period of acquisition is particularly interesting for the WKY rats. As mentioned above, session 4 is an average point where more WKY rats exhibit more avoidance responding than escape responding (Servatius et al., 2008; Beck et al., 2011; Jiao et al., 2011; Perrotti et al., 2013); however, session 4 is also proximal to when non-reinforced responding changes in WKY rats (Perrotti et al., 2013). This pattern was observed in the rats sacrificed following session 2 versus session 4, with the decrease in responding during the ITI occurring in both those with a FL-signaled and unsignaled ITI. Yet, neither reinforced nor non-reinforced behavior was correlated with c-Fos density measured in any of the three subregions of the mPFC. Moreover, lesions to either or both the PL and IL cortex failed to significantly affect the emitting of non-reinforced responses during the ITI period. Therefore, if the expression changes of c-Fos activation, which we observed in the PL cortex of male WKY rats, were caused by the acquisition of lever-press avoidance, it is not likely tied to changes in the specificity of responding (i.e., less during the ITIs). Instead, the analyses of the avoidance latencies suggest that proximal to the fourth session (when avoidance responses equate that of escape responses) the PL cortex is involved in driving the avoidance response to be emitted quicker. In contrast, the IL lesions do not affect the latencies, and the combined lesions only statistically affected latency during the fourth session. The PL cortex has been hypothesized as having a role for detecting threat to facilitate fear responses through the amygdala (Stern et al., 2010), but given the type of non-species-specific behavior required to avoid the shock, it may be that the PL cortex is driving the behavioral response through other pathways not specifically associated with the amygdala (Bravo-Rivera et al., 2014). Combined PL + IL lesions exhibited greater variability in avoidance latencies than the PL-lesions alone, but the trend was similar to that exhibited by those with lesions limited to the PL cortex. Thus, lesions of both PL and IL cortex suggest the combined activation of both cortices modulate the *rate and speed* by which lever-press active avoidance is acquired and emitted, but, at the same time, neither cortical area is *required* for that acquisition process.

These data are somewhat similar to shuttle-avoidance lesion studies in that damage to the PL cortex is reported to not be detrimental (Moscarello and LeDoux, 2013), but unlike that paradigm, it appears that only more diffuse PL and IL cortex lesions can significantly slow active-avoidance acquisition. This may be due to the type of response that is required, running versus lever-pressing, species-specific versus non-species-specific behavior. Still, c-Fos activation in PL cortex and IL cortex has been shown to be correlated with shuttle behavior alone and/or shuttling combined with freezing, respectively, in a non-cued Sidman avoidance procedure (Martinez et al., 2013). These c-Fos measures, however, occurred following a session where the aversive stimulus (shock) was absent, and, in that study, the average amount of shuttling decreased by nearly 50% during that shock-free session (Martinez et al., 2013). Thus, it cannot be ruled out that the activation measured in the brains of those rats may have been due to the new learning that the shock was not on the same temporal schedule. As mentioned above, recent inactivation studies suggest the IL cortex may be particularly critical for response inhibition in fear and avoidance paradigms (Bravo-Rivera et al., 2014; Sangha et al., 2014). The current data also suggests that increases in PL c-Fos may reflect a motivational drive to avoid; therefore, it is possible during extinction both PL and IL cortices may be activated as a re-evaluation of predictive threat occurs.

Another consideration that needs to be recognized is that the current study focused its efforts on understanding particular aspects of avoidance learning in an anxiety disorder vulnerability model. The WKY rats acquire active avoidance differently than SD rats and clearly differ in their ability to extinguish the lever-press avoidance behavior (Servatius et al., 2008; Beck et al., 2010, 2011; Jiao et al., 2011). Information pertaining to threat evaluation is transmitted from the basolateral amygdala (BLA) to PL cortex through a direct projection that changes its directional plasticity in response to stress (Maroun and Richter-Levin, 2003; Maroun, 2006). Those studies were conducted in SD rats, which are not as stress sensitive as WKY rats (Pare, 1989; Tejani-Butt et al., 1994; Bravo et al., 2011; Jiao et al., 2011). Therefore, it is entirely possible that these regions of the WKY mPFC are not functioning in the same manner as the male SD rats during avoidance learning. For example, latencies to respond to the warning signal with a lever-press avoidance response are quicker in male WKY rats versus male SD rats (Beck et al., 2010). Also, increases in anticipatory responding occur earlier in WKY rats versus SD rats, suggesting the processing of prospective threat may occur more readily in WKY rats (Perrotti et al., 2013). Thus, although the necessary processing of the FL ITI-signal does not appear to require the IL cortex, differences in other aspects of avoidance learning displayed between WKY and SD rats may be due to neurotransmission differences between the BLA and PL cortex. Amygdala – mPFC connectivity and functioning is documented to be different in humans with anxiety disorders, although the particular pattern of difference is still not resolved (Gilboa et al., 2004; Monk et al., 2006; McClure et al., 2007; Liberzon and Sripada, 2008; Etkin et al., 2010; Tromp et al., 2012; Demenescu et al., 2013; Stevens et al., 2013; Killgore et al., 2014). Therefore, the WKY rat can be a very useful model to study how *abnormal* prefrontal functioning can lead to avoidance susceptibility, avoidance extinction resistance, and overall anxiety vulnerability.

## Conclusion

The paradoxical behavioral inhibition and active-avoidance susceptibility demonstrated by WKY rats provide a unique opportunity to examine how an intact, albeit abnormal, brain can produce behaviors akin those expressed by individuals with pathological anxiety. Although we reaffirmed that male WKY rats more readily acquire active avoidance when a discrete signal is presented during the “safe” ITIs, the behavioral and neuroimmunohistological data do not readily support the hypothesis that the ITI signal acquires the properties of a “safety signal”. In fact, we observed brief exposures to the same stimulus can facilitate eyeblink conditioning in the male WKY rats, suggesting intermittent exposures to the FL may be serving to increase arousal, if it is novel. This may explain why the facilitation of active-avoidance learning is apparent in the early phases of training. Changes in the activation of the PL cortex occurring at the end of that early acquisition period may represent a change in how the rats were responding to the signals in the environment. Specifically, it appears to occur at a period when damage to the PL cortex is associated with longer avoidance latencies. Thus, the activation of the PL cortex, following the ITI-signal enhanced period, may represent the acquired association of threat to the elicitation of the avoidance behavior. Future work will be focused on understanding how the PL cortex contributes to the acquisition of active-avoidance learning, and whether abnormal neural activity in the PL cortex of WKY rats contribute to their avoidance-susceptible behavioral phenotype. This research, utilizing a behaviorally inhibited model, complements other recent work that has begun to dissociate functions of mPFC subregions in the human mPFC and the adoption of avoidance (Bzdok et al., 2013).

## Conflict of Interest Statement

The authors declare that the research was conducted in the absence of any commercial or financial relationships that could be construed as a potential conflict of interest.
